# Differential Distribution of Genes Encoding the Virulence Factor *Trans*-Sialidase along *Trypanosoma cruzi* Discrete Typing Units

**DOI:** 10.1371/journal.pone.0058967

**Published:** 2013-03-11

**Authors:** Juan M. Burgos, Marikena G. Risso, Simone Frédérique Brenière, Christian Barnabé, Oscar Campetella, María Susana Leguizamón

**Affiliations:** 1 Instituto de Microbiología y Parasitología Médica, Facultad de Medicina, Universidad de Buenos Aires, Buenos Aires, Argentina; 2 Unité de Recherche MIVEGEC, Maladies Infectieuses et Vecteurs: Ecologie, Génétique, Evolution et Contrôle, Institut de Recherche pour le Développement (IRD), Représentation de l'IRD en Bolivie, La Paz, Bolivia; 3 Instituto de Investigaciones Biotecnológicas, Universidad Nacional de San Martín, San Martín, Buenos Aires, Argentina; Centro de Pesquisa Rene Rachou/Fundação Oswaldo Cruz (Fiocruz-Minas), Brazil

## Abstract

*Trypanosoma cruzi* the agent of Chagas disease is a monophyletic but heterogeneous group conformed by several Discrete Typing Units (DTUs) named TcI to TcVI characterized by genetic markers. The *trans*-sialidase (TS) is a virulence factor involved in cell invasion and pathogenesis that is differentially expressed in aggressive and less virulent parasite stocks. Genes encoding TS-related proteins are included in a large family divided in several groups but only one of them contains *TS* genes. Two closely related genes differing in a T/C transition encode the enzymatically active TS (aTS) and a lectin-like TS (iTS). We quantified the *aTS/iTS* genes from TcII and TcVI aggressive and TcI low virulent strains and found variable *aTS* number (1–32) per haploid genome. In spite of being low TS enzyme-expressers, TcI strains carry 28–32 *aTS* gene copies. The intriguing absence of *iTS* genes in TcI strains together with the presence of aTS/iTS in TcII and TcVI strains (virulent) were observed. Moreover, after sequencing *aTS/iTS* from 38 isolates collected along the Americas encompassing all DTUs, the persistent absence of the *iTS* gene in TcI, TcIII and TcIV was found. In addition, the sequence clustering together with T/C transition analysis correlated to DTUs of *T. cruzi*. The consistence of *TS* results with both evolutionary genome models proposed for *T. cruzi*, namely the “Two Hybridization” and the “Three Ancestor” was discussed and reviewed to fit present findings. Parasite stocks to attempt genetic KO or to assay the involvement of iTS in parasite biology and virulence are finally available.

## Introduction

Chagas disease is a chronic disabling disease caused by the protozoon *Trypanosoma cruzi*. With an estimated 8–10 million people already infected, and about 40,000 new cases/year, Chagas disease represents a major public health, social and economic problem in Latin America where about 100 million people are at risk [1]. Besides the vectorial spread, blood transfusion, organ transplants and congenital transmission increase the worldwide dissemination risk due to migratory processes as in the USA where it has been estimated that 300,000 people are infected [2].

*T. cruzi* constitutes a monophyletic but genetically heterogeneous group. Based on various genetic markers and evolutive and population genetics interpretations of data, *T. cruzi* populations have been classified into six Discrete Typing Units (DTU) namely TcI to TcVI. Recently a seventh group sampled in bats and named TcBat has been added [3], [4]. Because of the predominantly clonal evolution of the parasite, these DTUs are rather stable in space and time, constituting a useful framework for epidemiological and evolutionary analysis [5]. This genetic diversity seems to be correlated with a geographical distribution [3], [6],[7] and with biological characteristics of the parasite such as culture growth, pathogenicity in mice, evolution in the insect vector, susceptibility to antichagasic drugs and tissular tropism in animal and human infections. Human infection displays different clinical evolutions ranging from asymptomatic to cardiomyopathy, megaviscera or even death. Different outcome incidences are also determined by host genetics, the presence of mixed infections, cultural factors, etc. [8]. Within the endemic area, heterogeneous geographical distribution of DTUs has been extensively described suggesting that the genetic composition of the parasite could be partly responsible for the different manifestations of Chagas disease. Broadly, TcI is found from the south of the USA in the sylvatic environment to northern South America where it seems to be responsible for chagasic cardiomyopathy [7], [9]–[11]. In Southern Cone countries, TcI is usually associated to the sylvatic cycle whereas TcII, TcV, and TcVI are relatively more abundant than TcI in the domestic cycle [3], [12], [13]. In this region, human infections present higher rates of severe heart affectation [7], [14]–[18] and digestive abnormalities, which are exceptional in northern South America and Central America [19], [20]. TcIII which is usually isolated from vectors and sylvatic reservoirs has a low prevalence in human infections [11], [21], [22] whereas TcIV shows a similar geographical distribution but higher incidence in human infection [15], [23]–[25].

Although sialic acid is crucial for the life cycle of *T. cruzi*, being involved in host cell adhesion/invasion processes and escape from the complement, the parasite is unable to synthesize this sugar *de novo*. To circumvent this gap, the parasite expresses the *trans*-sialidase (TS), that transfers α(2,3)-linked sialyl residues among glycoproteins or glycolipids. Circulating TS activity alters the sialylation pattern of the cellular glycoconjugates leading to hematological and immunological abnormalities associated to the disease [26]–[28].

Genes encoding TS are included in a large family composed of at least 1439 members [29], a figure certainly underestimated due to the expected collapse when assembling closely similar sequences. Although several different groups of genes can be discerned, only one of them includes those that code for the TS proteins [30], [31]. It has been estimated that as many as 150 genes of this group are included in the genome [32] where two TS isoforms, the active enzyme (aTS) and an enzymatically inactive TS (iTS) are encoded. Comparison of the aTS *vs.* iTS deduced amino acid sequences shows variations in 20 residues, although the inactivation is entirely due to the single crucial Tyr_342_His replacement as a consequence of a T/C transition [33]. The replacement by histidine renders the protein enzymatically inactive but allows retaining the substrate binding ability conferring therefore a lectin-like activity [32], [34]. This strongly suggests a physiologic role for iTS in parasite attachment to substrates or cell surface receptors that might explain its conservation. Crystallographic analyses and enzyme kinetic assays [35] have recently shown that iTS retains residual hydrolytic activity. By using the recombinant iTS, a co-stimulating host T-cells effect have been adscribed [36].

Previous efforts to associate parasite genetic classification and biological features have allowed us to determine the expression/shed of aTS as a marker of pathogenicity that segregates strains belonging to different lineages [37]. In this study our aim was to analyze the distribution of genes encoding the virulence factor TS among DTU-representative isolates collected along the Americas in the context of their evolution. We found *aTS* in all analyzed stocks and the striking absence of *iTS* genes in TcI, TcIII and TcIV DTUs. The consistence of the TS results with current *T. cruzi* evolutionary genome models was reviewed to fit findings. Parasite stocks to attempt genetic KO or to assay the involvement of iTS in parasite biology and virulence are now available.

## Materials and Methods

### Trypanosoma cruzi isolates

The study was carried out in a panel of 38 parasite isolates encompassing all DTUs (nine TcI stocks, seven TcII, two TcIII, five TcIV, six TcV, and nine TcVI) obtained from various ecological origins (vectors, animal reservoirs and human infections) spanning all the endemic area from Argentina to the USA.

### *Trypanosoma cruzi* genomic DNA purification

DNA from Ac, Hc, K-98, SN, Br, CMA, ChVal, HE, HT, RA, Q501/3, Tulahuen, ML, Alf, FAL and Cvd parasite strains was obtained from peripheral blood trypomastigotes. DNA from Silvio X10, Tu18, M5631, Can III, CL Brener, CID, H1, QUE, CBBcl2, ESMcl3Z2, IVVcl4, MAS1cl1, MVBcl8, X109/2, 3.1, 92122102R, STC10R, STC16Rcl1, MNcl2, SC43cl1, CA15, P63cl1 strains was obtained from epimastigotes. The Blood and Cell Culture DNA Purification Kit (Qiagen) or conventional phenol-chloroform DNA extraction methods were used.

### DTU characterization

All *T. cruzi* DNA samples were genotyped using polymerase chain reaction (PCR) strategies following Burgos et al [17] algorithm of classification. Some *T. cruzi* stocks (CID, H1, QUE, CBBcl2, ESMcl3Z2, IVVcl4, MAS1cl1, MVBcl8, X109/2, 3.1, 92122102R, STC10R, STC16Rcl1, MNcl2, SC43cl1, CA15, P63cl1) were also characterized by MLEE [38]–[40] for DTU assignment.

For PCR characterization, five reactions targeted to the intergenic region of spliced leader genes (SL-IR), 24sα rDNA and the A10 fragment were carried out on each DNA sample to determine the parasite DTU. The PCR amplicon size for each DTU was: Tc I: SL-IRac (150 bp), SL-IR-I (475 bp), and 24sα HnPCR (140 bp); TcII: SL-IRac (157 bp), SL-IR-II (425 bp), 24sα HnPCR (140 bp) and A10-PCR (690 bp); TcIII: SL-IRac (200 bp), 24sα HnPCR (125 bp), and A10-PCR (630 bp); TcIV: SL-IRac (200 bp) and 24sα HnPCR (140/145 bp); TcV: SL-IRac (157 bp), SL-IR-II (425 bp), 24sα HnPCR (125 or 125+140 bp), and A10-PCR (630 bp); TcVI: SL-IRac (157 bp), SL-IR-II (425 bp), 24sα HnPCR (140 bp), and A10-PCR (630 bp).

### Quantification of *aTS* and *iTS* genes

To assess the number of genes per haploid genome coding for aTS and iTS in each parasite isolate, a real-time PCR-based strategy was applied. These assays were performed at the facilities of Eurofins Medigenomix GmbH (Germany) on an ABI 7900 HT Sequence Detection System (Applied Biosystems) using the universal mix of ABI TaqMan reagents (Applied Biosystems). Briefly, the region containing the mutation was amplified with the following flanking oligonucleotides: 5'-TGGGCAAGTATCCATTGGTGATG-3' and 5'-TGATCTCATGCAAACAGTACAGCTT-3'. In the same reaction, a pair of fluorescent-labeled probes specific for the two possible sequences at the mutation position (5′-AATTCCGCCTACAGCT-3′ coupled to FAM to detect *aTS* and 5′-AAAATTCCGCCCACAGCT-3′ coupled to VIC which binds to *iTS*) allowed the quantification of both genes. The analysis was normalized by quantification of the *T. cruzi* pyruvate dehydrogenase (PVDH)-encoding gene present as a single copy per haploid genome [41]. This reaction included a set of primers (5'-CGGCGTACCAGCCTGAGAT-3' and 5'-ACCTGAAGGCCCGGAATG-3') and a labeled probe (5'-TACCGTCGTGGCGACT-3') that hybridizes the *pvdh* gene.

Plasmids containing the *aTS* or *iTS* genes were used as control and during test standardization. In the data analysis, the intensity of each signal was a definite value (Ct, cycle of threshold), which is inversely related to the amount of complementary DNA. The proportion of both genes was calculated for each *T. cruzi* isolate as the average of two independent determinations.

### Amplification and sequencing of *TS* genes

Two upstream and two downstream primers to the region containing the T/C transition of TS-encoding genes were designed (5′ primers: TS-51, 5′-GGAGGCTGTCGGCACGCTCTC-3′ and TS-5, 5′-GCTTCACTGCCGTGACCATCG-3′; 3′ primers: TS-31, 5′-TCACGCAGCGGTACGCATCCT-3′ and TS-3, 5′-CAGCGGGACCACAACCACGCT-3′), so that all the TS sequences annotated in the GenBank were targeted in at least one of the four PCR reactions to be performed. Amplification was carried out with 0.4 µM of each primer, 5 U of *Pfu* polymerase enzyme (Promega), 2.5 mM of dNTPs, and 100 ng of genomic *T. cruzi* DNA as template in 50 µl final reaction volume. The PCR cycle consisted of 30 rounds of 94°C for 45 s, 63°C for 45 s, and 72°C for 45 s, with a first step of 2 min at 94°C and one last step of 5 min at 72°C. PCR products were analyzed by electrophoresis in 2% agarose gel. Amplicons were purified and both strands sequenced with the primers used for amplification. Chromatograms were visually examined to determine the presence of C and/or T in the first position of the codon 342. Firstly TS-51/TS-31 primer set was used and those genomes rendering only the Tyr_342_ codon were then subjected to the other three PCR reactions. The IUPAC nomenclature for the genetic code was used to define single nucleotide polymorphism (SNP) positions with mixed base identification set to 15% of the highest peak. Sequences were deposited in the GenBank with the accession numbers KC286514, KC286515, KC286516, KC286517, KC286518, KC286519, KC286520, KC286521, KC286522, KC286523, KC286524, KC286525, KC286526, KC286527, KC286528, KC286529, KC286530, KC286531, KC286532, KC286533, KC286534, KC286535, KC286536, KC286537, KC286538, KC286539, KC286540, KC286541, KC286542, KC286543, KC286544, KC286545, KC286546, KC286547, KC286548, KC286549, KC294586 and KC294587.

### Sequence comparison of the region around the codon 342

A 455-bp consensus sequence of each *T. cruzi* stock was obtained by comparison of forward and reverse sequences of the TS-51/TS-31 PCR reactions. Sequences from the different stocks were aligned and compared by using ClustalW2 program [42]. A clustering tree was built by using SplitsTree4 [43] with the following options: i) the “UncorrectedP” method which computes the proportion of positions at which two sequences differ was used; ii) the ambiguous state codes (such as W, M, V …) were handled with the option ‘Average’ meaning that the contribution at a site is averaged over all possible resolutions of the ambiguous codes, with the exception that sites having the same ambiguous code contribute zero; iii) the distance-based method used was UPGMA because we considered that evolution rate must be the same upon all branches; the bootstrap was conducted with 1,000 iterations.

## Results

### Quantification of *aTS* and *iTS* genes in the genome of *T. cruzi*

To analyze the distribution of TS-encoding genes in the genome of parasites analyzed by Risso et al [37], we performed a quantitative analysis of *aTS* and *iTS* by real-time PCR on DNA samples. The single copy *Pvdh* gene [41] was included as internal reference to standardize the number of haploid genome copies in the test. Primers that amplify the region containing the single nucleotide polymorphism (SNP) that determines the loss of enzymatic activity were used together with two probes that differ in only one base (T/C transition) and comprise the Tyr codon (to hybridize *aTS* genes) or His codon (complementary to the sequence of *iTS* genes), respectively. No cross-recognition between the aTS and iTS probes under test conditions was found, as assayed with plasmids harboring the corresponding gene. No Ct could be determined with the *iTS*-complementary probe for low-virulence TcI strains, indicating no detection of *iTS* genes carrying the T/C transition. As shown in Table 1, the genome of these *T. cruzi* isolates harbors 28 to 32 copies of aTS-coding genes. On the other hand, data obtained from the aggressive strains RA, Cvd, CL Brener and the clone Q501/3 (all belonging to TcVI) and Br (TcII) indicated that both the *aTS* and *iTS* genes were present with high variability in the gene copy number (Table 1). In these genomes, the *aTS/iTS* rate was high for TcII (3/1) and quite balanced in TcVI (2/1 and 1/1).

**Table 1:** Quantification of *aTS* and *iTS* in parasites representing high and low TS activity producers.

T. cruzi		pvdh	aTS		iTS	
DTU	Isolate^a^	Ct	Ct	Gene copies	Ct	Gene copies
**TcI**	**Ac**	39.004±0.606	23.907±0.146	30 (100%)	ND	0
	**Hc**	34.172±0.098	18.473±0.001	32 (100%)	ND	0
	**K-98**	36.529±0.186	22.679±0.102	28 (100%)	ND	0
**TcII**	**Br**	18.269±0.022	16.912±0.032	3 (75%)	17.611±0.029	1 (25%)
**TcVI**	**RA**	28.925±0.018	14.767±0.065	28 (64%)	20.900±0.877	16 (36%)
	**Q501/3**	20.465±0.155	17.717±0.066	4 (67%)	19.043±0.048	2 (33%)
	**Cvd**	17.791±0.094	17.074±0.378	1 (50%)	17.867±0.159	1 (50%)
	**CL Brener**	29.132±0.161	14.719±0.032	29 (60%)	19.494±0.141	19 (40%)

a) Parasites correspond to low (TcI) and high (TcII and TcVI) TS activity producer stocks as described by Risso *et al* [37].

Parasite DNA was subjected to quantitative real time PCR and *aTS*/*iTS* presence was determined by using probes labeled with reporter dyes. Gene number per haploid genome was determined by Ct comparison with that obtained for the *pvdh* single copy gene. Ct: cycle of threshold; ND: not detectable.

### Presence of the T/C SNP in codon 342 of *TS* genes

**Figure 1:**
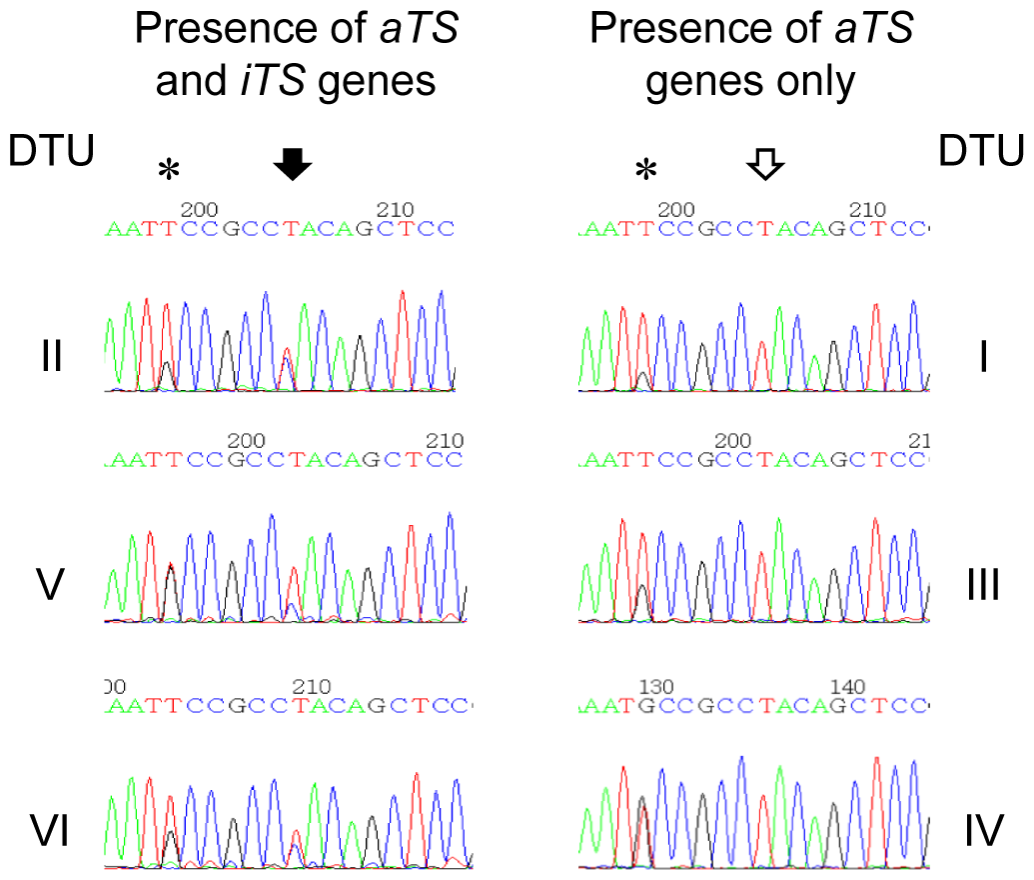
Chromatograms from the region flanking the T/C SNP. Sequencing examples from parasites belonging to the six DTUs are shown. Black arrow points T and C nucleotides in TcII, TcV and TcVI PCR products. Empty arrow points the same position in TcI, TcIII and TcIV amplicons, where only T was observed. Star indicates a T/G SNP (K in IUPAC code) present in all tested parasites.

The findings presented above strongly suggest the intriguing absence of *iTS* genes in TcI parasite genome. To further test the differential distribution of *aTS* and *iTS* among *T. cruzi* stocks belonging to different DTUs, the T/C SNP was directly searched by sequencing PCR-amplified gene fragments comprising the surrounding region. To assess the representation of the Tyr_342_His mutation among these different parasite stocks, two primers were designed upstream and two other downstream the target codon. All *TS* genes deposited at GenBank were covered given that all these known sequences were targeted at least once in the PCR strategy designed. Table 2 summarizes the biological sources and geographical origins of the parasite isolates tested and the findings observed. Notably, whereas all stocks from the DTUs TcII, TcV and TcVI contained both *aTS* and *iTS* genes (with T and C in the first position encoding codon 342 observed as a mixed peak in the chromatograms, see Figure 1), TcI, TcIII and TcIV parasites tested depicted only T (corresponding to *aTS* genes) indicating the absence of genes coding for iTS in agreement with results shown in Table 1.

**Table 2:** Origin and DTU classification of parasite isolates with TS isoforms predicted presence.

*T. cruzi* isolate	Source	Country^a^/Area	DTU	TS^b^
Ac	Human	Ar/Chaco	I	aTS
Hc	Human	Ar/unknown	I	aTS
SN	Human	Ar/Misiones	I	aTS
K-98	Human	Ar/San Luis	I	aTS
FAL	Human	Ar/Chaco	I	aTS
Silvio X10	Human	Br/Para	I	aTS
CID	Human	Me/Oaxaca	I	aTS
H1	Human	Me/Yucatan	I	aTS
QUE	Vector^c^	Me/Queretaro	I	aTS
Br	Human	Ar/unknown	II	aTS/iTS
Tu18	Vector^d^	Bo/Tupiza	II	aTS/iTS
CBBcl2	Human	Ch/Region IV	II	aTS/iTS
IVVcl4	Human	Ch/Region IV	II	aTS/iTS
MVBcl8	Human	Ch/Region IV	II	aTS/iTS
ESMcl3	Human	Br/Bahia	II	aTS/iTS
MAS1cl1	Human	Br/Fed. District	II	aTS/iTS
M5631	Armadillo^e^	Br/I. Marajo	III	aTS
X109/2	Dog	Pa/Pte. Hayes	III	aTS
Can III	Human	Br/Belem	IV	aTS
3.1	Vector^d^	Pe/Arequipa	IV	aTS
92122102R	Raccoon	USA/Georgia	IV	aTS
STC10R	Raccoon	USA/Georgia	IV	aTS
STC16Rcl1	Raccoon	USA/Georgia	IV	aTS
CMA	Human	Ar/Chaco	V	aTS/iTS
ChVAL	Human	Ar/unknown	V	aTS/iTS
HE	Human	Ar/Chaco	V	aTS/iTS
HT	Human	Ar/Chaco	V	aTS/iTS
MNcl2	Human	Ch/Region IV	V	aTS/iTS
SC43cl1	Vector^d^	Bo/Chuquisaca	V	aTS/iTS
RA	Human	Ar/Chaco	VI	aTS/iTS
Q501/3	Human	Ch/Region IV	VI	aTS/iTS
Cvd	Human	Ar/Mendoza	VI	aTS/iTS
ALF	Human	Ar/Chaco	VI	aTS/iTS
ML	Human	Ar/unknown	VI	aTS/iTS
Tulahuen	Human	Ch/Region IV	VI	aTS/iTS
CL Brener	Vector^d^	Br/RG do Sul	VI	aTS/iTS
CA15	Vector^d^	Bo/Santa Cruz	VI	aTS/iTS
P63cl1	Vector^d^	Pa/Pte. Hayes	VI	aTS/iTS

a) Ar: Argentina; Br: Brasil; Me: México; Bo: Bolivia; Ch: Chile; Pe: Perú; Pa: Paraguay. b) TS isoforms predicted presence. aTS: active *trans*-sialidase; iTS inactive *trans*-sialidase. c) unidentified blood-sucking vector; d) *Triatoma infestans* (vector bug). e) *Dasypus novemcinctus*.

### Sequence comparison of *TSs* amplicons

**Figure 2:**
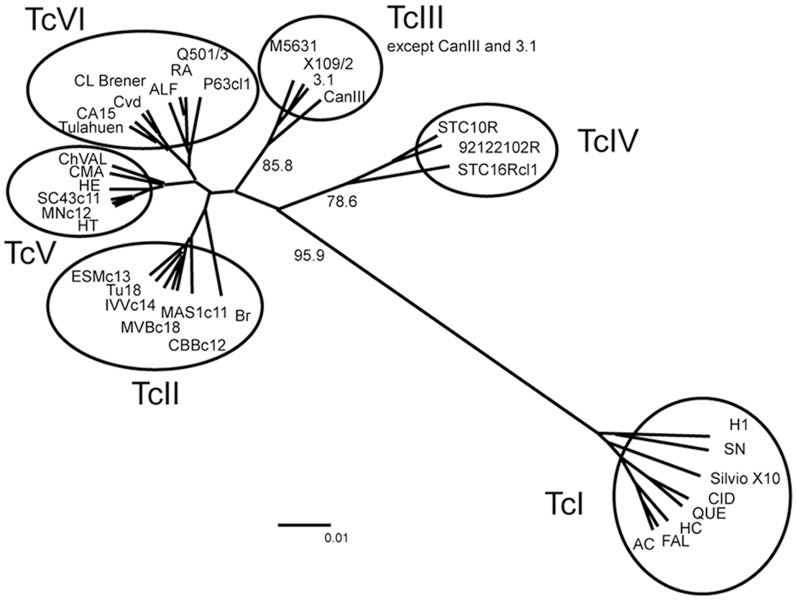
UPGMA tree based on TS genes sequence alignment (with ambiguous states). Each circle grouped all 38 *T. cruzi* strains in their respective previous assigned DTU, except CAN III and 3.1 that were previously assigned to TcIV. Significant bootstrap values for TcI, TcIII and TcIV are reported, bootstrap values for other DTUs were <50.

To further analyze the region in search for other SNPs that might be useful to classify parasites, we compared the 455 bp TS gene region sequenced from the 38 *T. cruzi* stocks belonging to the six DTUs. Besides the nucleotide position corresponding to the T/C transition (Figure 1), sequences also include the TGG codon for Trp_312_, one of the two aromatic residues that participate in substrate binding [35]. Interestingly, all stocks from every DTU encoded the Trp_312_ codon sequence in that position (see Figure S1). On the other hand, sequences revealed several differences among the six DTUs as other SNPs beyond the T/C transition. Nine SNPs were synapomorphs of DTUs or groups of DTUs. Based on these SNPs and other less conserved polymorphisms (asterisks in Figure S1), the sequence clustering analysis as an UPGMA tree (Figure 2) showed that parasite strains were differentiated into six groups that coincide with the previous DTU assignment, TcI, TcII, TcIII, TcIV, TcV and TcVI with only two exceptions (CANIII and 3.1 strains which are classified as TcIV but here clustered with TcIII). However, bootstrap values were remarkably significant for TcI, TcIII and TcIV and lower (<50) for the other DTUs.

## Discussion

The *T. cruzi* current classification into six principal DTUs, mainly based on genetic characteristics, proved to be a valid framework to study the biological variability of *T. cruzi* that is widely recognized [3]. Indeed genetic diversity of the parasite is undoubtedly an important factor influencing many biological parameters (reviewed in [3]), and it has been suggested that it could be partly responsible for the different clinical outcomes of Chagas disease [8]. No strong correlation has yet been observed between the development of pathology and parasite's DTUs. However, most clinical and epidemiological studies in human infections associate TcI with infections in patients living from Colombia northwards, whereas TcII, TcV and TcVI have been detected as the most prevalent etiological agents at the south of South America, where Chagas disease presents high rates of severe heart affectation [15]–[18]. In particular, digestive syndromes, also observed in southern countries, appear to be associated only to TcII, TcV and TcVI parasites [19], [20]. Although *T. cruzi* populations display differential virulence and pathogenic characteristics, genetic markers linked with the evolution of the infection and their outcomes have not been identified to date. Different efforts have been made to find virulence factors that correlate with the current parasite classification [37], [44]–[46]. A recent study [47] has shown differential expression of 29 out of 261 proteins that are overexpressed in *T. cruzi* stocks belonging to a given DTU. We have previously shown [26], [37] that virulent parasites currently studied that belong to TcII or TcVI, express/shed more TS activity than the less aggressive TcI stocks. In addition, increased circulating TS activity correlates with several abnormalities observed early during the infection [26], [28]. The involvement of shed aTS in the alterations of the histoarchitecture of the spleen, thymus and ganglia, as well as in the induction of thrombocytopenia has been evaluated by both the administration of recombinant aTS to naive mice and the neutralization of the enzymatic activity during the acute infection [26], [28], [48]. Here the fine quantitative SNP mapping allowed us to identify *aTS/iTS* differences that remain hidden in genomic analyses [49] because *iTS* genes are not included in databases taken as reference. The present quantification of *TS* genes in parasite stocks exposed the absence of *iTS* genes together with the presence of similar copy number of *aTS* in all TcI parasite stocks tested (28 to 32 copies/haploid genome). On the other hand, variable aTS and iTS copy number (from 1 to 29 and 1 to 19 copies/haploid genome, respectively) were found in TcII and TcVI. These DTUs showed an *aTS/iTS* ratio that ranged from 1 to 3 (Table 1). These current observations allow us to conclude that the actual protein expression is independent of the number of *aTS* genes because genomes from high aTS producer parasites contain similar or even lower *aTS* gene copy numbers than those from TcI parasites with little production of aTS [37]. Moreover, the absence of *iTS* genes in this group raises the possibility of a correlation between this gap and the lower virulence previously observed for the TcI parasites assayed [37]. Considering that the aggressive strains [37] contain genes encoding iTS isoform, a role for this protein in the virulent behavior could be inferred. The analysis of *iTS/aTS* genes was then extended to representative parasite stocks encompassing the six DTUs, isolated from several sources (insect vectors, animal reservoirs and human infections) in different geographical areas (from the USA to Argentina). We found that *aTS* genes were present in all 38 parasite populations, emphasizing the central role of this enzyme in parasite biology. It is worth noting that *iTS* was observed exclusively in stocks from DTUs TcII, TcV and TcVI but intriguingly absent in all TcI, TcIII and TcIV stocks analyzed. The absence of cumulated mutations or stop codons in iTS sequences, together with the fact that we have always found the same T/C transition that encodes the Tyr_342_His amino acid replacement as the enzyme inactivation mechanism, indicate that the same *iTS* genes, conserved among all the TcII, TcV and TcVI parasite populations, are probably expressed. The Trp_312_ and Tyr 119 codons that are crucial in creating the two-aromatic residue-stacking site for the galactosyl portion of the substrate [50] are also conserved in aTS and iTS proteins from all DTUs. In support, a residual enzyme activity has been recently found for iTS protein [35] emphasizing that it has similar properties to aTS in sequence and folding. Furthermore, *in vitro* assays have demonstrated the co-stimulatory properties of iTS proteins on the immune system [36]. The strong sequence conservation in all *iTS* genes supports that iTS plays an evolutionary selectable role, instead of representing just a collection of pseudogenes. Therefore, an involvement in parasite attachment/invasion to host cells can be postulated because iTS acts as a lectin, able to bind not only small oligosaccharides but also sialylated glycoproteins [32], [34], a relevant feature in the physiological scenario of parasite infection.

Interestingly, our findings also reveal the existence of parasites with highly reduced TS genes content that provide models to develop genomic KO, a largely expected tool to extend the study of the biological relevance of TS whose generation has been hampered by the high gene copy numbers always reported for TS. Moreover, the ongoing transfection assays with the *iTS* gene might provide with a nice opportunity to test the actual relevance of iTS in parasite biology and pathogenesis.

**Figure 3:**
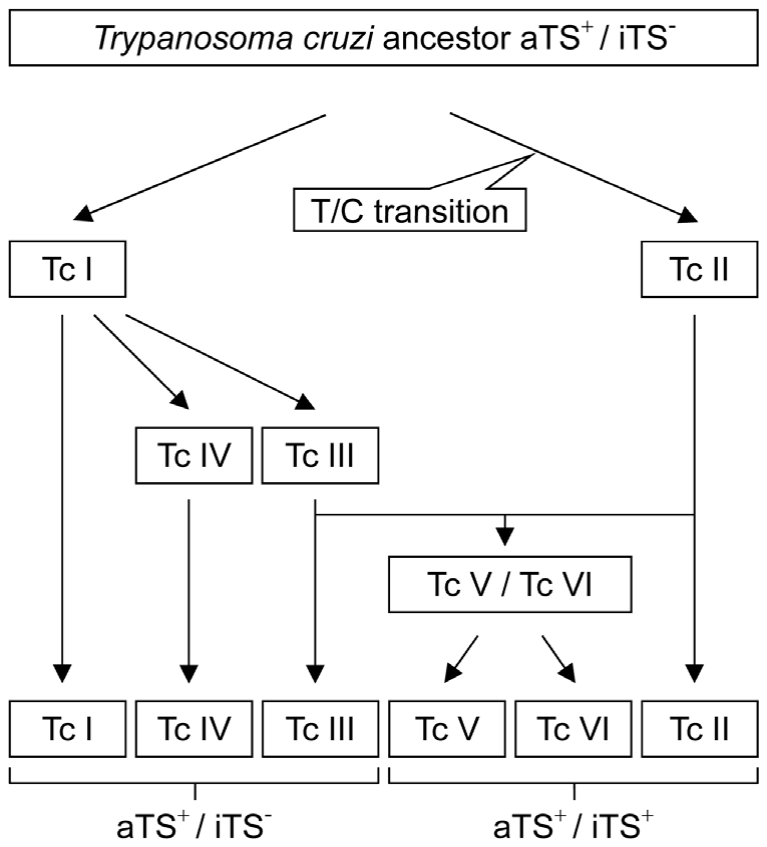
Parasite DTU evolution model proposed. Considering the previously proposed evolution models [3] together with data reported here, an evolution model is drawn where the acquisition of the *iTS* gene by a single mutation event by TcII places TcI and TcII as the only ancestors for all the other DTUs. A single hybridization event of TcIII and TcII derivates in TcV and VI as previously proposed.

In 2009, an expert committee revised the information available about *T. cruzi* evolution and clustering. They remember that the partition of *T. cruzi* in six principal DTUs could be explained by two alternative models for their origin: the ‘Two Hybridization’ model giving rise to TcIII and then to TcV and TcVI through hybridization of two ancestors (TcI and TcII) [51] and the ‘Three Ancestor’ where the ancestors TcI, TcII and TcIII gave rise to the hybrids TcV and TcVI [52]. The current distribution of aTS/iTS suggests a closer relationship of TcI with TcIII-TcIV than with the other DTUs as well as a related evolution of TcII, TcV and TcVI. Indeed, the sequence analysis that reflect the variability of a set of genes coding for the same virulence factor (TS) fits with the six DTUs clustering, although TcII, TcV and TcVI DTU were not supported by significant bootstrap values because the hybrid nature of TcV and TcVI, reduces the bootstrap values, and if these strains are removed from the analysis (see Figure S2), TcI and TcII DTUs are everyone very well supported by high bootstrap value (93.8 and 98.3 respectively), and TcIII and TcIV are grouped together with a lower bootstrap value (60.9). However, this group is further divided into two clusters, one including CanIII, M5631, X109/2 and 3.1 strains (bootstrap value of 95.8) and the other comprising STC16Rcl1, STC10R and 92122102R strains (bootstrap value of 71.4). Although several scenarios can explain the current variability of the TS genes within DTUs, considering that TcI and TcII are ancestors [51], [52] and that *iTS* may have originated from *aTS* genes through a single mutation event, the common ancestor of TcI and TcII should not have had *iTS*. After *iTS* consolidation in TcII, its delivery during subsequent hybridization events could explain its presence in TcV and TcVI parasites (newest hybrid groups [51], [52]). Further, considering the “Two Hybridization” model, the absence of *iTS* in TcIII and TcIV could be explained by an inequitable ancient recombination, gene conversion or by loss of *iTS* corresponding genes. In the “Three Ancestor” model [52] TcIII-TcIV could have early diverged from TcI and propagated without *iTS* genes. The close relationship between TcIII and IV with TcI is also supported by findings with cruzipain and TSSA antigens [45], [53]. As shown in Figure 3, an alternative picture of *T. cruzi* evolution might be drawn that fits the previously obtained data plus that reported here. Ancestor parasites lack *iTS*, then TcII acquired *iTS* and both TcI and TcII became ancestors of all the other DTUs. A single hybridization event is postulated between TcII and TcIII that rendered TcV and VI, TcIII and IV seem to have evolved from TcI instead from hybridization of TcI with TcII because this hypothesis requires two events, the hybridization itself followed by the lost of the *iTS* genes contributed by TcII genome.

Finding an association between clinical manifestations and parasite genotype is a difficult task. The multiclonal nature of most natural infections and the histotropic behavior of different parasites lead to partial characterizations when bloodstream and/or other infected tissue samples are analyzed [54], [55]. The regional diversity of Chagas disease outcomes has been attributed to a set of complex interactions where the parasite genetic makeup, as well as the environmental and the host immunogenetic background are some of the factors involved (reviewed by [56]). In the challenge to identify links between the infecting DTUs and the pathogenesis induced by *T. cruzi* we presented for the first time the differential distribution among parasite populations of *iTS/aTS*, a virulence factor-related gene that is well correlated with the evolutionary history of the parasite. The expression of this complex (aTSa/iTS) of virulent genes may be a key to better understand the mechanism of virulence and its relationship with *T. cruzi* evolution.

## Supporting Information

Figure S1 **Consensus sequence of** ***TS*** **gene internal region.** Sequence alignment of *T. cruzi* stocks encompassing the 6 DTUs (TcI to TcVI). (.): conserved sites; (▾): SNPs that identify a group of parasites (inter-DTU polymorphism). In those positions, depicted nucleotide for each DTU was present in all sequences obtained from all parasites of each DTU (named as IUPAC code); (*): other polymorphic positions not shared by all stocks within a DTU (intra-DTU polymorphisms); TGG: Trp_312_ codon conserved in all stocks from all DTUs; Box: Tyr_342_His codon where Thymidine (encoding Tyr) and Cytosine (encoding His) are present in all stocks belonging to TcII, TcV and TcVI whereas only Thymidine was found in TcI, TcIII and TcIV genomes. No other mutations were found in this codon.
(TIF)

Figure S2 **UPGMA tree based on TS genes sequence alignment (with ambiguous states) not including hybrid DTUs.** To avoid deviations induced by the hybrid nature of *T. cruzi* TcV and TcVI DTUs, UPGMA tree was built excluding these DTUs.
(TIF)
